# Supplementary material for: Combined Immunoinformatics to Design and Evaluate a Multi-Epitope Vaccine Candidate against Streptococcus suis Infection
Source: Vaccines (Basel). 2024 Jan 29;12(2):137. doi: 10.3390/vaccines12020137 (PMC10892848; doi:10.3390/vaccines12020137)
Supplement: Supplementary file 1 [file vaccines-12-00137-s001.zip › vaccines-2806073-supplementary.pdf]

Table S1 Primers used in this study

Primers	Sequence（5'-3'）	Function	Reference
MVSS-F	GGATCCGGGCCAGGTCCCGGAT	A fragment for recombinant	This study
MVSS-R	GAGCTCGAGTCGTGTTCCAGCG	protein MVSS gene	
T7-F	TAATACGACTCACTATAGGG	A fragment for plasmid	
T7-R	TGCTAGTTATTGCTCAGCGG	pET-28a gene	
GAPDH-F	TGCACCACCAACTGCTTAG	A fragment for murine	[1]
GAPDH-R	GGATGCAGGGATGATGTTC	housekeeping marker gene	
IL-1β-F	TCCAGGATGAGGACATGAGCAC	A fragment for murine IL-1β	
IL-1β-R	GAACGTCACACACCAGCAGGTTA	gene	
IL-6-F	CCACTTCACAAGTCGGAGGCTTA	A fragment for murine IL-6	
IL-6-R	GCAAGTGCATCATCGTTGTTCATAC	gene	
TNFα-F	GGTGCCTATGTCTCAGCCTCTT	A fragment for murine TNFα	
TNFα-R	GCCATAGAACTGATGAGAGGGAG	gene	
IL-2-F	GCGGCATGTTCTGGATTTGACTC	A fragment for murine IL-2	
IL-2-R	CCACCACAGTTGCTGACTCATC	gene	
IL-10-F	CGGGAAGACAATAACTGCACCC	A fragment for murine IL-10	
IL-10-R	CGGTTAGCAGTATGTTGTCCAGC	gene	

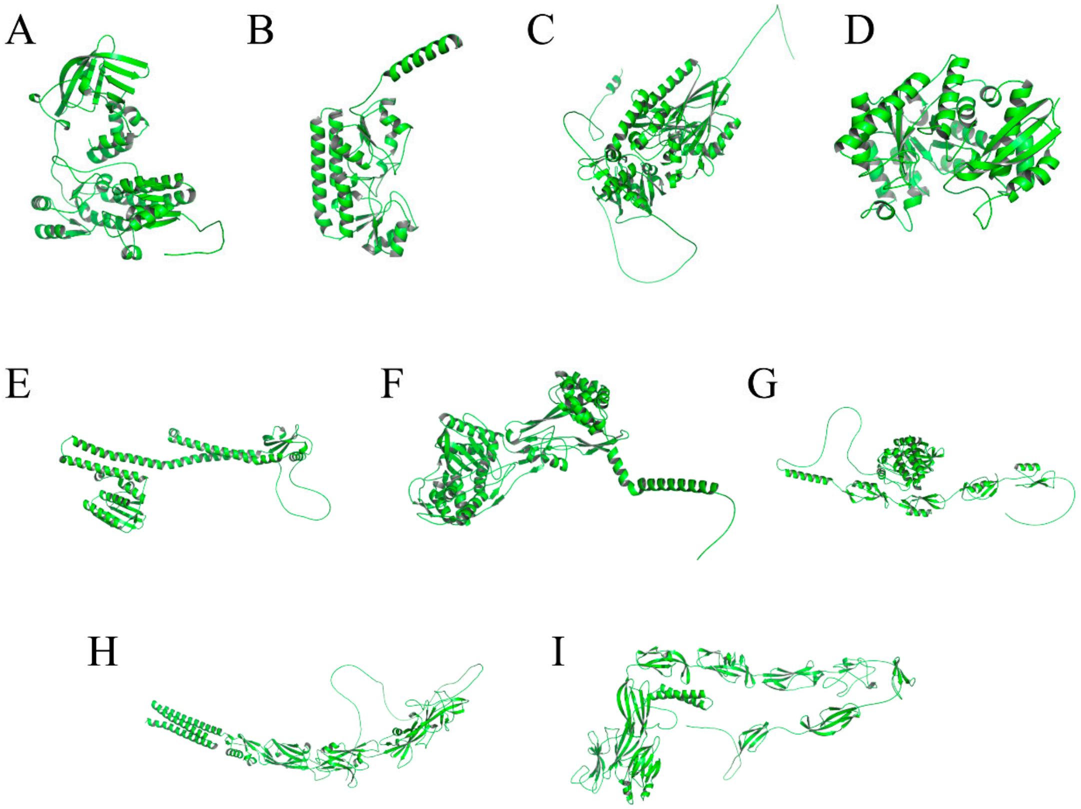

Figure S1 Three-dimensional modeling of candidate proteins. (A) CP024974_1_00171, (B) CP024974_1_00407, (C) CP024974_1_01634, (D) enolase, (E) CP024974_1_01033, (F)

CP024974_1_00497, (G) CP024974_1_00699, (H) extracellular protein factor (I) muramidase-released protein

A **MVSS:**GPGPG*LRMKLPKS***GEEEHEGHDHSEEGHSHAYD**GPGPG*LRMKLPKS***GDTAGTTTDTKTPEKANDGG**GPGPG*LRMKLPKS***GEHEAVELRDGDKSRYLGLG**GPGPG*LRMKLPKS***IAGYRTVNSDGTKTETVEET**GPGPG*LRMKLPKS***EKGVNAIVVLAHVPATSKDG**GPGPG*LRMKLPKS***LNILFSIVIFLFLVLILRLA**GPGPG*LRMKLPKS***TTPGTNGEVPNIPYVPGYTP**GPGPG*LRMKLPKS***AKEAGYTAVVSHRSGETEDS**GPGPG*LRMKLPKS***TKDGLRYVLVPSKTDGEENG**GPGPG*LRMKLPKS***NGPRTEINMKKRKNKPLEHD**

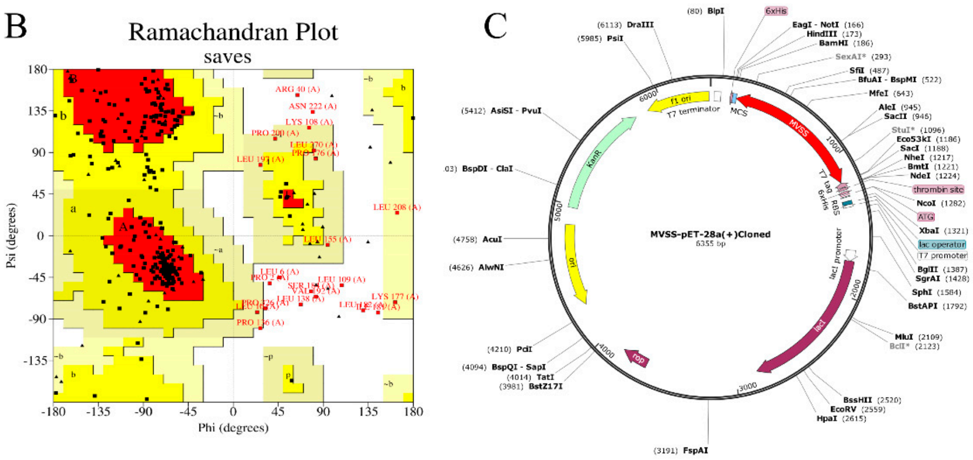

Figure S2 MVSS amino acid sequence, modeling evaluation and recombinant plasmid presentation. The 10 epitopes were connected using the linkers "GPGPG" and "LRMKLPKS" following the idea that hydrophilicity increases from the center outward. (B) Ramachandran diagram of the modified MVSS structure. Red regions: most favorable [A, B, L]; yellow regions: additional allowances [a,b,l,p]; light yellow regions: generous allowances [~a, ~b, ~l, ~p]. (C) Illustration of MVSS forming recombinant plasmid with pET28a(+) vector.

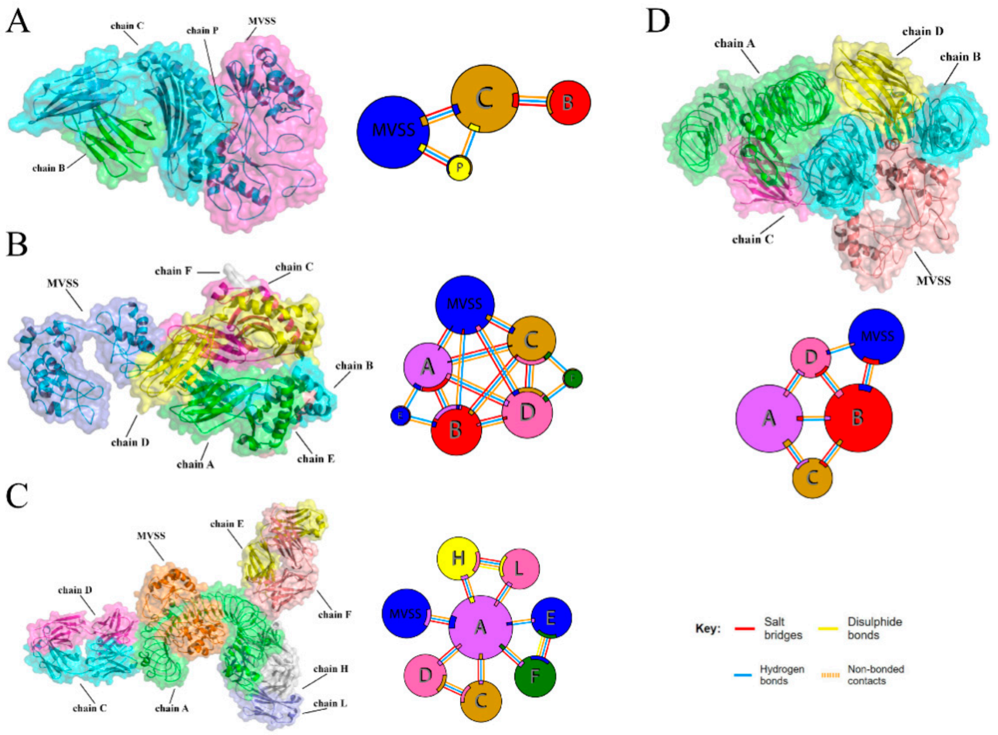

Figure S3 Molecular docking of MVSS and human immune receptors. Schematic illustration of interaction power between MVSS-MHC I (A), MVSS-MHCII (B), MVSS-TLR 3 (C) and MVSS-TLR4 (D) complexes.

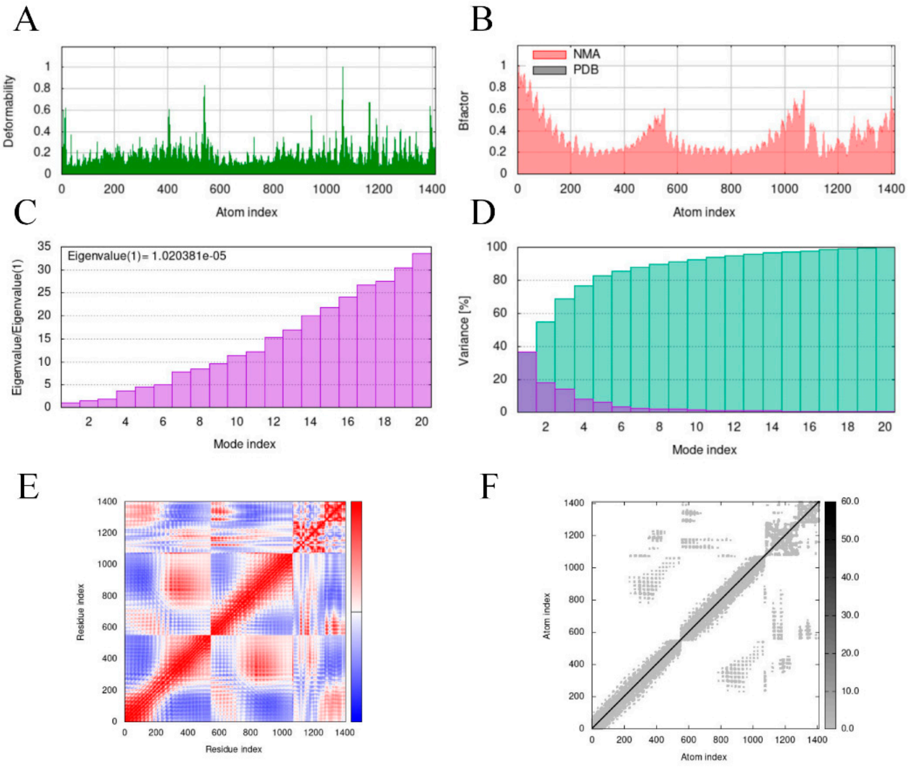

Figure S4 Molecular dynamics simulations of the MVSS-TLR2 complex. (A) Deformability map of the docking complex. (B) Calculation of the B-factor by NMA. (C) Eigenvalues of docked complexes. (D) NMA variance. (E) Docking complex amino acid residue atom pair covariance matrix plot. (F) Docking complex elastic network model connection spring diagram.

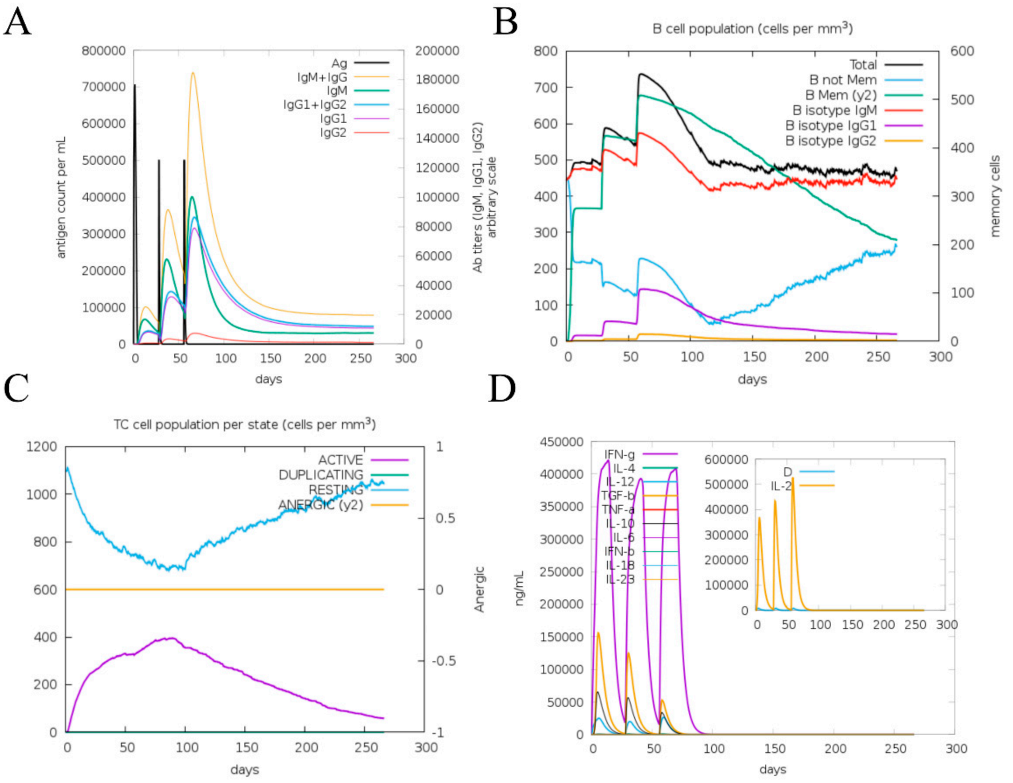

Figure S5 Simulation of the immunological response of the host following immunization with the multi-epitope vaccine *in silico*. (A) Significantly elevated antibody levels after vaccination with the multi-epitope vaccine. (B) B cells and memory immunoglobulin levels rise following immunization. T-lymphocyte (C) and cytokine (D) numbers rose after vaccination and persisted at elevated levels throughout the exposure period.

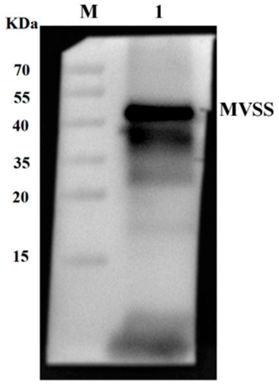

Figure S6 Western blot analyses of MVSS. M indicated Marker, lane 1 meant western blot to detect the reactivity of anti-rMVSS serum with rMVSS.

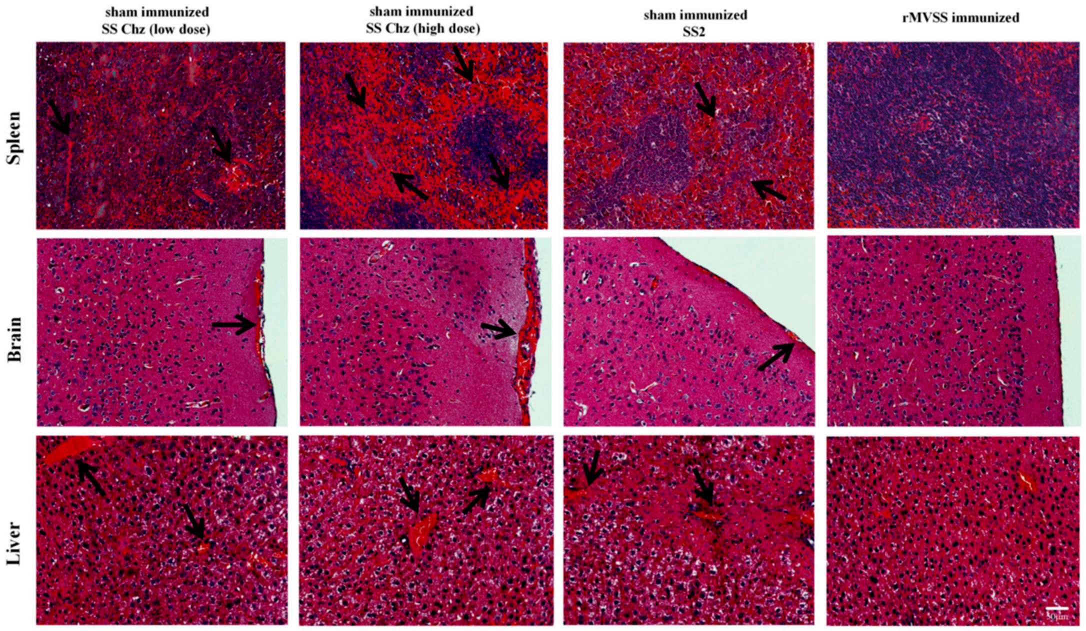

Figure S7 Histopathological damage assessment of spleen, brain and liver tissues of rMVSS-immunized and sham-immunized mice after being challenged by various serotypes of *S. suis*. Black lines indicated bleeding areas.

Reference

[1] Zhang, Y.; Liang, S.; Zhang, S.; Zhang, S.; Yu, Y.; Yao, H.; Liu, Y.; Zhang, W.; Liu, G. Development and Evaluation of a Multi-Epitope Subunit Vaccine against Group B Streptococcus Infection. Emerg. Microbes Infect. 2022, 11, 2371–2382, doi:10.1080/22221751.2022.2122585.
